# Best practice exercise for emerging depression in multiple sclerosis: A systematic review and meta-analysis

**DOI:** 10.1177/02692155241262884

**Published:** 2024-07-25

**Authors:** Kristiina Ahola, Diana Dorstyn, Nicole Prideaux

**Affiliations:** 1 Faculty of Health and Medical Sciences, School of Psychology, The University of Adelaide, Adelaide, Australia; 2 Faculty of Health and Medical Sciences, School of Allied Health Sciences and Practice, The University of Adelaide, Adelaide, Australia

**Keywords:** Multiple sclerosis, rehabilitation, depression, systematic review

## Abstract

**Objective:**

To examine the effects of instructor-led exercise on depression symptoms in adults with multiple sclerosis, with a focus on moderating factors to treatment response.

**Data sources:**

Cochrane Library, Embase, PEDro, PsycINFO and PubMed databases were searched until 21 April 2024.

**Review methods:**

The reporting quality of included studies assessed (PEDro and TESTEX scales). Hedges’ *g* effect sizes were calculated and pooled using random and mixed-effects modelling.

**Results:**

Twenty-two independent studies (*k*), representing 785 participants with relapsing remitting or progressive MS, were included. Individual studies varied in their reporting quality (PEDro range: 3–8) and did not routinely detail exercise parameters (TESTEX range: 5–13). Nonetheless, exercise reduced core symptoms of depression (*g*_w _= .52, CI: .30–.73, *P *< .01). Treatment effects were, however, not maintained once training had ceased (*g*_w _= −.53, CI: −.80 to .24, *P *≤ .01, *k *= 5). Both aerobic and non-aerobic exercise groups experienced a significant (*P *< .01) reduction in depression scores. Larger gains were noted by those with better ambulation at baseline (*P *= .03).

**Conclusion:**

Regular exercise can help to stabilise mood for people living with multiple sclerosis, regardless of session frequency or duration. Treatment efficacy could be maximised by addressing potential barriers for those with limited mobility, including exercise type, delivery and intensity. Protocol registered on Open Science Framework [https://osf.io/zfymq/].

The benefits of regular exercise for adults diagnosed with multiple sclerosis, a demyelinating central nervous system disease, are well established. Exercise training offers a safe lifestyle treatment^1–3^ that can build physical endurance and reduce the risk of cognitive decline.^4–6^ Exercise can also be useful for those with concurrent mild to moderate symptoms of depression^7^; a common neuropsychiatric manifestation which can worsen disease course.^8–10^ Current guidelines do, however, recommend guided exercise prescription across the broad spectrum of multiple sclerosis.^11–14^ Therapist oversight is particularly crucial for those experiencing low motivation due to depression.^10^

The isolated effect of exercise on depression in multiple sclerosis is, however, compromised by study methodology. Trials have typically incorporated exercise training as one component of a lifestyle intervention,^7^ or compared its effectiveness to other active treatments.^5,15^ Exercise prescription is a further consideration. Whilst high ‘dose’ programs involving greater physical work have demonstrated benefits,^16^ community multiple sclerosis samples have not consistently reported a dose-response effect with exercise – at least not for depression.^17^ Even studies with healthy adults have reported mixed findings regarding the relationship between exercise duration or frequency and depression symptom severity.^18,19^ The comparative effectiveness of aerobic exercise for cardiorespiratory fitness and non-aerobic training for muscular strength, endurance, flexibility and/or fitness goals also warrants examination. There is evidence that both have positive psychological effects – although this may depend on degree of disability.^20–22^

The publication of randomised controlled trials in recent years offers an opportunity to re-examine high-quality evidence on the mental health effects of exercise in multiple sclerosis. The current review therefore provides an up-to-date meta-analysis to address the following research questions: (a) in adults with multiple sclerosis, what are the effects of physical exercise on depression symptoms compared to no treatment or usual care? and (b) do sample (e.g., age, disease severity) and exercise parameters (e.g., duration and frequency) influence treatment effectiveness?

## Methods

### Study identification, eligibility and screening

As per our protocol [Open Science Framework on 4th July, 2023; https://osf.io/zfymq/], The Cochrane Library, Embase, PEDro, PsycINFO and PubMed databases were searched from inception until 18 May 2023 using terms that were compiled with the assistance of an expert research librarian (see Table S1, Online Supplementary Material). An updated, time-limited search was then completed prior to publication (on 21 April 2024). The reference lists of included studies and previous reviews^16,17,20–22^ were additionally searched, with one unique study identified through this process.^23^

The first author screened all records using Covidence systematic review software (Veritas Health Innovation). The second author then screened 50% of records (6742 titles and abstracts, 3969 full texts), with high inter-rater reliability at each stage (agreement > 90%; κ =.85^24^). The few discrepant articles were resolved through consensus discussion.

In addition to being published in the English language, or with English translation, studies had to involve the following criteria:

*Population*: Adult participants (*≥*18 years) with a self-reported or physician-confirmed diagnosis of multiple sclerosis.

*Intervention*: An instructor-led exercise program delivered individually, or as a group, to promote physical activity.^11–14,25^ Studies that supplemented in-class training with prescribed low-intensity, home-based exercise to help maintain or improve fitness were eligible. The use of standard gymnasium equipment (e.g., treadmills, ergometers) was acceptable, although the intervention had to involve real-time practice with a trained health professional.^11^ Exercise interventions that were self-managed or involved the use of specialised equipment and techniques (e.g., functional electrical stimulation, neuromodulation, robot assistance) were excluded as the focus of this review was on the direct effects of supervised exercise. Vestibular programs, a specialised form of physiotherapy to manage dizziness or balance issues, were also ineligible as were programs that either did not involve general exercise (e.g., pelvic floor muscle training), integrated exercise into the multi-disciplinary management of multiple sclerosis, or involved exercise testing but no intervention per se.

*Comparison:* Only randomised controlled studies that used a passive control condition (i.e., wait list or usual care, but no specifically assigned physiotherapy or psychosocial intervention) were included.

*Outcome*: Depression symptom severity was evaluated using a validated self-reported or clinician-administered measure, as a primary or secondary outcome, pre- and post-exercise.

*Time:* Studies needed to provide effect size data for short-term (pre to immediately post) and/or longer-term (post to follow-up) exercise effects on depression.

### Data extraction, preparation and analysis

This review followed the updated Preferred Reporting Items for Systematic Reviews and Meta-analyses guidelines.^26^ The first author extracted key study characteristics (e.g., sample size, demographic and MS data, effect size data). Details relating to exercise programs were also extracted in accordance with the Template for Intervention Description and Replication Checklist (TIDier^27^) and checked for accuracy by the second and third authors. Some data conversion was necessary: subgroup means were averaged, 95% confidence intervals converted to standard deviations, and medians with interquartile ranges converted to means (as per^28,29^).

The methodological quality of each study was evaluated using the Physiotherapy Evidence Database (PEDro) Scale (www.pedro.org.au, in press). Ratings were compared to confirmed scores for each study available on the PEDro database. Inter-rater reliability was high (98% accuracy). Post-hoc, we added an extension of PEDRO specifically developed for exercise trials; the Tool for the assEssment of Study qualiTy and reporting in Exercise (TESTEX^30^). Good item similarity was demonstrated between both scales (*r *= .79, *P *< 0.001).

Publication bias was assessed using a funnel plot. In the absence of publication bias, the plot represents a symmetrical triangle with equal spread of studies on either side.^31^ Asymmetry was quantified using Duval and Tweedie's trim and fill method, which estimates the number of unpublished (imputed) studies,^32^ and Egger's regression test.^33^

Standardised mean group differences (Hedges’ *g*) for each study were analysed using Comprehensive Meta-Analysis software (Version 4; Biostat) with an imputed intra-study correlation of *r *= .70. Outlier effects were first identified using a one-study removed sensitivity analysis. Effect estimates were then grouped by depression measurement and individual *g*'s weighted by their study's inverse variance prior to pooling (*g*_w_); positive values indicated greater improvement (i.e., lowered depression) with exercise training compared to controls. Additionally, *P* values were used to infer statistical significance of *g* and 95% CIs computed to estimate the precision of each *g*/*g*_w_. Between-study variance was quantified using tau (estimated *SD* of *g*), tau^2^ (variance of true effects), *I*^2^ (expressed as a percentage) and prediction intervals (i.e., the range of true effects that can be expected in future similar studies^34^). A random-effects (DerSimonian and Laird) model was used for these calculations to account for the clinical heterogeneity of MS, range of exercise programs and different depression measures used.

Univariate meta-regressions and subgroup analyses (using a *Q*-test based on analysis of variance and a mixed effects model^35^) were conducted to examine the association between treatment effects and key principles of exercise prescription for the general adult population^25^ namely, weekly session frequency (≤2 vs. ≥3 sessions) and total number of sessions within a program. The differential impact of aerobic, non-aerobic or programs that involved both modalities was also examined. Studies that evaluated both aerobic and non-aerobic exercise with the same control group were excluded from this analysis to meet the requirements for data independence.^34^ Finally, the contribution of disability severity (as measured by the ordinal Expanded Disability Status Scale or its surrogate, the Patient Determined Disease Steps^36,37^) and baseline depression scores were also explored.

## Results

**Figure 1.**
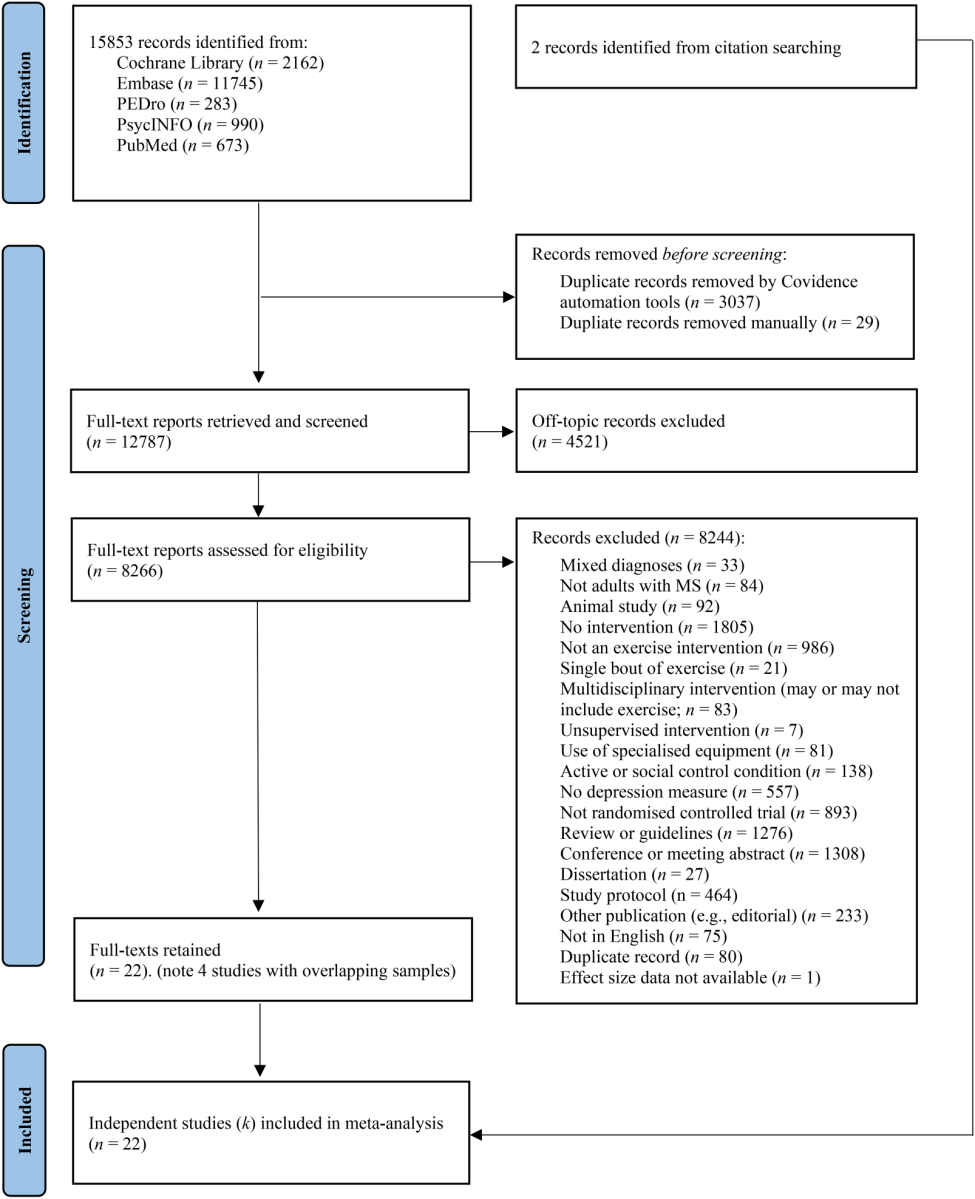
Flow chart of study selection process, adapted from PRISMA.^26^

Database searches yielded 15,853 records, of which 12,787 potentially relevant records were screened (see Figure 1). A further 8266 full texts were re-screened against the eligibility criteria. During the screening process, four papers with overlapping samples were identified,^38–42^ with the initial publication being retained.^39,40^ The final sample comprised 22 independent trials (Table 1^23,39,40,42–60^).

**Table 1.** Study characteristics.

Lead author (date) [reference]	Country	Total ^a^	% Female	Mean age (*SD*)	MS duration (*SD*)	Mean EDSS/ PDSS (*SD*), criterion or range	MS subtype	Exercise type	Exercises	Duration	Dropout ^b^	Control condition
Ahmadi et al (2013)^39^	Iran	31	100%	35.16 (9.01)	5.09 (4.09)	2.22 (1.16)	-	1) Aerobic 2) Non-aerobic	1) Treadmill 2) Yoga	3 sessions/wk 1) 30 min 2) 60–70 min 8 weeks	-	Waitlist
Baştürk et al. (2024)^42^	Turkey	31	55%	45.61 (11.22)^d^	13.16 (5.89)^d^	<6	-	Aerobic	Line dancing	2 sessions/wk 60 min 4 weeks	40%	Waitlist
Briken et al. (2014)^43^	Germany	46	-	49.80 (7.83) ^c^	15.75 (7.34) ^c^	4.95 (0.84) ^c^	74% SPMS 26% PPMS	1) Aerobic 2) Aerobic 3) Aerobic	1) Arm cycle 2) Rowing 3) Bicycle	22 sessions 15–45 min 8–10 weeks	1) 16.6% 2) 8.3% 3) 8.3%	Waitlist
Buttolph et al. (2023)^44^	United States	20	75%	44.0 (9.8)	9.9 (6.9)	-	90% RRMS 5% SPMS 5% Unknown	Non-aerobic	Qigong	1 session/wk 60–90 min 10 weeks	40%	Usual activities
Çakit et al. (2010)^45^	Turkey	30	-	36.05 (10.42) ^c^	8.18 (4.31) ^c^	<6	-	Mixed	Bicycle & Balance	2 sessions/wk 8 weeks	6.7%	Usual activities
Correale et al. (2021)^46^	Italy	27	100%	46.0 (7.2) ^c^	14.6(6.9) ^c^	2.25 (0.8)	100% RRMS	Mixed	Bicycle or Treadmill	2 sessions/wk 45–60 min 12 weeks	0%	Usual care
Dalgas et al. (2010)^47^	Denmark	38	-	48.42 (9.29) ^c^	7.37 (5.90) ^c^	3.80 (0.89) ^c^	100% RRMS	Non-aerobic	Resistance	2 sessions/wk 12 weeks	15.8%	Waitlist
Fleming et al. (2019)^48^	Ireland	12	100%	52.34 (7.06) ^c^	-	<3	-	Non-aerobic	Pilates	2 sessions/wk 60 min 8 weeks	40%	Waitlist
Hebert et al. (2011)^49^	United States	26	85%	46.4 (10.37) ^c^	7.1 (5.89) ^c^	-	88% RRMS 12% SPMS	Mixed	Bicycle & Stretching	2 sessions/wk 45 min 6 weeks	0%	Waitlist
Hortobágyi et al. (2022) ^50^	Hungary	40	90%	46.57 (6.31) ^c^	13.58 (4.11) ^c^	5.25 (0.23) ^d^	62% RRMS	1) Aerobic 2) Non-aerobic	1) Treadmill 2) Balance	25 sessions 1 h 5 weeks + 3 sessions/wk 2 years	-	Usual care
Kocica et al. (2022) ^51^	Czech Republic	46	83%	39.65 (8.27)	6.95 (4.37)	2.18 (0.70)	100% RRMS	Mixed	Circuit	1 session/wk 60 min 12 weeks	-	Usual activities
Langeskov- Christensen et al. (2021)^52^	Denmark	86	60%	44.8 (9.38) ^c^	9.75 (7.07)^c^	2.7 (1.5)	87% RRMS 7% PPMS 6% SPMS	Aerobic	Rowing, Bicycle, Cross Trainer	2 sessions/wk 30–60 min 24 weeks	9.5%	Waitlist
Learmonth et al. (2011) ^53^	Scotland	32	72%	51.55 (7.91) ^c^	13.1 (6.97) ^c^	6.02 (0.44) ^c^	-	Mixed	Circuit	2 sessions/wk 45–60 min 12 weeks	25%	Usual activities
Miller et al. (2011)^54^	Scotland	30	63%	54.6 (7.83)	15.85 (8.95)	7.05 (0.66) ^c^	37% PPMS 63% SPMS	Non-aerobic	Strength & Flexibility	2 sessions/wk 60 min 8 weeks	0%	Usual care
Najafi et al. (2023)^40^	Malaysia & Iran	56	100%	38.0 (5.46)	9.40 (5.61)	2.55 (1.16)	100% RRMS	1) Non-aerobic 2) Non-aerobic	1) Pilates 2) Yoga	3 sessions/wk 50–70 min 8 weeks	1) 25% 2) 21%	Usual activities
Negaresh et al. (2019) ^55^	Iran	66	66%	31.19 (3.03) ^c^	7.35 (4.04) ^c^	1.64 (1.05) ^c^	100% RRMS	Aerobic	Bicycle	3 sessions/wk 42–66 min 8 weeks	5.6%	No intervention
Oken et al. (2004) ^56^	United States	69	93%	49.02 (9.03) ^c^	-	3.08 (1.83) ^c^	-	1) Aerobic 2) Non-aerobic	1) Bicycle 2) Yoga	1 session/wk 90 min 6 months	1) 28.6% 2) 15.4%	Waitlist
Petajan et al. (1996) ^57^	United States	54	67%	39.96 (2.11) ^c^	7.61 (2.05) ^c^	3.31 (0.54) ^c^	-	Aerobic	Arm & Leg cycle	3 sessions/wk 40 min 15 weeks	22%	Waitlist
Savšek et al. (2021)^58^	Slovenia	28	82%	41.0 (6.25) ^c^	11.6 (6.19) ^c^	3.21 (1.51) ^d^	100% RRMS	Aerobic	Aerobics	2 sessions/wk 40–60 min 12 weeks	14.3%	Usual activities
Schulz et al. (2004) ^24^	Germany	28	43%	-	11.4 (1.6)	2.3 (0.2)	68% RRMS 7% PPMS 18% SPMS	Aerobic	Bicycle	2 sessions/wk 8 weeks	-	Waitlist
Sutherland et al. (2001) ^59^	Australia	22	55%	46.32 (4.87) ^c^	6.59 (4.61) ^c^	< 5.0	-	Mixed	Aquatics & Resistance	3 sessions/wk 45 min 10 weeks	-	Usual activities
Tollár et al. (2020) ^60^	Hungary	40	92%	46.39 (6.34) ^c^	13.57 (4.21)^c^	5–6	65% RRMS 35% PPMS	1) Aerobic 2) Non-aerobic	1) Bicycle 2) Balance	5 sessions/wk 60 min 5 weeks	1) 0% 2) 0%	Waitlist

*M*: mean; *SD*: standard deviation; F: female; EDSS: Expanded Disability Status Scale; RRMS: relapsing remitting MS; PPMS: primary progressive MS; SPMS: secondary progressive MS; (–): data not provided.

a Number of participants enrolled at baseline.

b Number of exercise participants who dd not complete final assessment.

c Estimated from median value and interquartile range.^29^

d Subgroup means combined.^28^

### Sample characteristics

The pooled sample of 785 persons had an average age of 44 years (*SD* = 6.04) and were living with mild to moderate levels of disability for at least 10 years (*SD* = 3.34). Participants were recruited via multiple sclerosis societies, social media and outpatient databases. Most had experienced relapses of neurological symptoms, aside from Miller et al.^54^ who targeted those with a progressive disease course. Most also identified as female (74%), consistent with the 3:1 ratio of females to males diagnosed.^61^ Additional illness characteristics (i.e., use of disease-modifying treatments) were not routinely reported.

Depression symptom severity was self-reported, more commonly with the original Beck Depression Inventory and its later revision. Measures that are sensitive to treatment change, namely the Inventory of Depressive Disorders and Quick Inventory of Depressive Symptomology, also featured.^43,48^ Notably, studies did not typically feature participants that met clinical criteria for a depressive disorder.

### Exercise programs

Twenty-seven exercise programs were evaluated across the 22 studies (Table 1). Most studies evaluated aerobic exercise to maximise heartrate (e.g., treadmill training, bicycle ergometry, dancing^24,42,43,52,55,57,58^) as opposed to non-aerobic activities to build strength, flexibility and body awareness (e.g., yoga, pilates^40,44,47,48,54^). Mixed-modalities, combining both aerobic and non-aerobic exercise in the one program also featured.^45,46,49,51,53,59^

Exercise sessions were typically delivered in a rehabilitation gym or clinical setting with certified instructors (e.g., physical therapists, physiotherapists). There were exceptions to this: an online, supervised yoga and pilates sessions delivered shortly after COVID-19,^40^ a home-based program with physiotherapy visits,^54^ and a selection of community qigong classes.^44^ The small group format (e.g., 4–6 per group^50,51^) was preferred, although studies did not always make this explicit. Programs were also tailored to meet the individual needs and restrictions of each person.^40,42,43,45,49,53^ Session duration ranged from 15 to 90 mins (*M *= 1 hour, *SD *= 14.52) scheduled two to three times per week over 4–24 weeks (*M *= 10 weeks, *SD *= 5.17). The exception was Hortobágyi et al.'s^50^ long-term comparative effectiveness trial which involved five weeks of high-intensity and high-frequency cycling, or balance exercises followed by a two-year maintenance program. Individual studies encouraged regular daily self-practice (e.g., 5–10 min daily^44^), with Oken et al.,^56^ even offering an exercise bicycyle for home use. Exercise diaries were additionally used to keep track of fitness goals.^48,60^

Exercise intensity was formally evaluated by measuring age-predicted peak heartrate,^39,49,58^ the Borg Rating of Perceived Exertion Scale^39,49^ or maximum effort on a resistance movement.^43^ Self-regulated intensity – such as choosing when to increase the speed on a treadmill or how long to hold a yoga pose – was also encouraged.^39,48,53,56^

Aside from a minor ankle strain^49^ and discomfort with Qigong leg/arm movements,^44^ exercise was well tolerated. Reasons for withdrawal were largely unrelated to the exercise itself (e.g., relapse of symptoms, medication side effects, mild ill health, transportation difficulties, scheduling conflicts^42,48,53,56^). Briken et al.^43^ identified fatigue as an issue for some participants, despite tailoring their training schedule to aerobic fitness level.

Control participants were encouraged to continue their normal physical activity or usual care.^40,44–46,50,51,53,54,58,59^ Others were waitlisted and allocated to the exercise program upon study completion.^24,39,42,43,47–49,52,56,57,60^

### Risk of bias assessments

PEDro and TESTEX ratings are listed in Tables 2 and 3. Methodological concerns included the challenges of concealing group allocation (from participants, therapists, assessors) due to the physical nature of the interventions examined. Intention-to-treat analysis was also not routinely adopted, despite being the ‘gold standard’ for the interpretation of randomised controlled trials.^62^ Similarly, reporting of exercise elements varied widely among the interventions (Table 3).

**Table 2.** PEDro ratings within and between studies.

Lead author (date) [reference]	1. Eligibility criteria*	2. Random allocation	3. Concealed allocation	4. Baseline comparability	5. Blinding of subjects	6. Blinding of therapists	7. Blinding of assessors	8. Adequate follow-up	9. Intention- to-treat	10. Between-group comparisons	11. Point estimates & variability	TOTAL
Ahmadi et al. (2013) ^39^	1	1	0	1	0	0	0	1	1	1	1	6
Baştürk et al. (2024) ^42^	1	1	0	1	0	0	1	0	0	1	1	5
Briken et al. (2014) ^43^	1	1	1	1	0	0	0	1	1	1	1	7
Buttolph et al. (2023) ^44^	0	1	1	0	0	0	0	0	0	0	1	3
Çakit et al. (2010) ^45^	1	1	0	1	0	0	1	0	0	1	1	5
Correale et al. (2021) ^46^	0	1	0	1	0	0	1	1	0	1	1	6
Dalgas et al. (2010) ^47^	1	1	0	1	0	0	0	1	0	1	1	5
Fleming et al. (2019) ^48^	1	1	1	1	0	0	0	0	0	0	1	4
Hebert et al. (2011) ^49^	1	1	1	1	0	0	1	1	1	1	1	8
Hortobágyi et al. (2022) ^50^	0	1	1	1	0	0	1	1	0	1	1	7
Kocica et al. (2022) ^51^	0	1	0	1	0	0	0	0	0	1	1	4
Langeskov-Christensen et al. (2021) ^52^	0	1	1	1	0	0	0	0	1	1	1	6
Learmonth et al. (2011) ^53^	1	1	0	1	0	0	1	1	1	1	1	7
Miller et al. (2011) ^54^	1	1	1	1	0	0	1	1	0	1	1	7
Najafi et al. (2023) ^40^	1	1	0	1	0	0	0	0	0	1	1	4
Negaresh et al. (2019) ^55^	1	1	1	1	0	0	1	1	0	1	0	6
Oken et al. (2004) ^56^	1	1	0	1	0	0	1	0	0	1	0	4
Petajan et al. (1996) ^57^	1	1	0	1	0	0	0	1	0	1	1	5
Savšek et al. (2021) ^58^	1	1	0	1	0	0	1	1	0	1	1	6
Schulz et al. (2004) ^24^	0	1	0	1	0	0	0	1	0	1	1	5
Sutherland et al. (2001) ^59^	0	1	0	1	0	0	0	1	0	1	1	5
Tollár et al. (2020) ^60^	1	1	1	1	0	0	1	1	1	1	1	8
% Meeting Criterion	68	100	41	95	0	0	50	64	27	91	91	6

0 = criterion not met, 1 = criterion met.

* Not included in total score.

**Table 3.** TESTEX ratings within and between studies.

Lead author (date) [reference]	1. Eligibility criteria	2. Random allocation	3. Concealed allocation	4. Baseline comparability	5. Blinding of sassessor	6. Adequate follow-up	7. Intention- to-treat	8. Between- group comparisons	9. Point estimates & variability	10. Control group activity monitoring	11. Relative intensity constant	12. Exercise volume & expenditure	TOTAL
Ahmadi et al. (2013) ^39^	1	1	0	1	0	1	1	1	1	0	0	1	8
Baştürk et al. (2024) ^42^	1	1	0	1	1	1	0	2	1	0	0	0	8
Briken et al. (2014) ^43^	1	1	1	1	0	2	1	1	0	0	1	1	10
Buttolph et al. (2023) ^44^	0	1	1	0	0	2	0	1	1	0	0	0	5
Çakit et al. (2010) ^45^	1	1	0	1	1	1	0	1	1	0	1	1	8
Correale et al. (2021) ^46^	1	1	0	1	1	3	0	1	1	0	1	1	11
Dalgas et al. (2010) ^47^	1	1	0	1	0	2	0	1	1	0	0	1	8
Fleming et al. (2019) ^48^	1	1	1	1	0	2	0	1	1	0	0	0	8
Hebert et al. (2011) ^49^	0	1	1	1	1	2	1	2	1	0	1	1	12
Hortobágyi et al. (2022) ^50^	1	1	1	1	1	1	0	0	1	1	1	1	10
Kocica et al. (2022) ^51^	1	1	0	1	0	0	0	1	1	0	0	0	5
Langeskov-Christensen et al. (2021) ^52^	0	1	1	0	0	1	1	1	1	0	1	1	8
Learmonth et al. (2011) ^53^	1	1	1	1	1	0	1	1	1	1	0	0	9
Miller et al. (2011) ^54^	1	1	1	1	1	3	0	1	1	0	0	0	10
Najafi et al. (2023) ^40^	1	1	0	0	0	0	0	1	1	1	0	0	5
Negaresh et al. (2019) ^55^	1	1	1	1	1	3	0	1	1	0	1	1	12
Oken et al. (2004) ^56^	1	1	0	1	1	2	0	1	1	0	0	0	8
Petajan et al.(1996) ^57^	1	1	0	1	0	3	0	1	1	0	1	1	10
Savšek et al. (2021) ^58^	1	1	0	0	1	2	0	1	1	0	1	1	9
Schulz et al. (2004) ^24^	1	1	0	1	0	0	0	1	0	0	1	1	6
Sutherland et al. (2001) ^59^	1	1	0	0	0	1	0	1	1	0	0	0	5
Tollár et al. (2020) ^60^	1	1	1	1	1	3	1	1	1	0	1	1	13
% meeting criterion	86	100	45	77	50	82	27	95	91	14	50	55	9

0 = criterion not met; 1 = criterion met, with additional points awarded for items 6 (maximum score of 3) and 8 (maximum score of 2).

**Figure 2.**
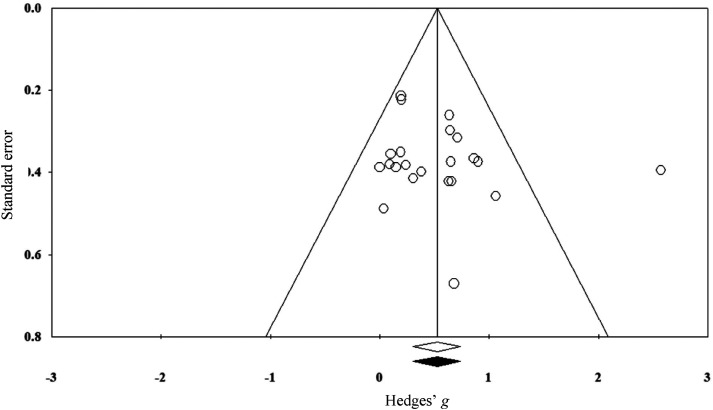
Funnel plot for primary meta-analysis indicating no imputed studies.

A funnel plot analysis suggested a low likelihood of publication bias, with most studies evenly scattered around the mid-point. This was confirmed by the trim-and-fill method, which revealed no imputed studies (see Figure 2), and Egger's non-significant regression test (intercept = 1.34; CI: −1.22 to 3.91; *P *= 0.28).

A one-study removed sensitivity analysis revealed no statistical outliers: the pooled *g*_w_ remained statistically significant even after removing individual studies.

### Effectiveness of exercise training

Pooled findings are presented in Table 4, with individual effect sizes grouped by depression measurement and time frame provided in Table 5. A positive trend was seen across all studies, with seven trials reporting immediate and significant improvements in depression scores with exercise.^39,40,43,47,51,55,57^ Programs involving ergometer exercise training protocols produced the largest effects.^43,57^ The effectiveness of Hatha yoga, which incorporates postures, meditation and breathing methods, was also demonstrated.^39,40^ Equally effective were circuit training performed at moderate intensity,^51^ and resistance training with active rest periods.^47^ The pooled prediction interval did, however, show a wider range of expected treatment effects – including null or negative effects (Table 4).

**Table 4.**
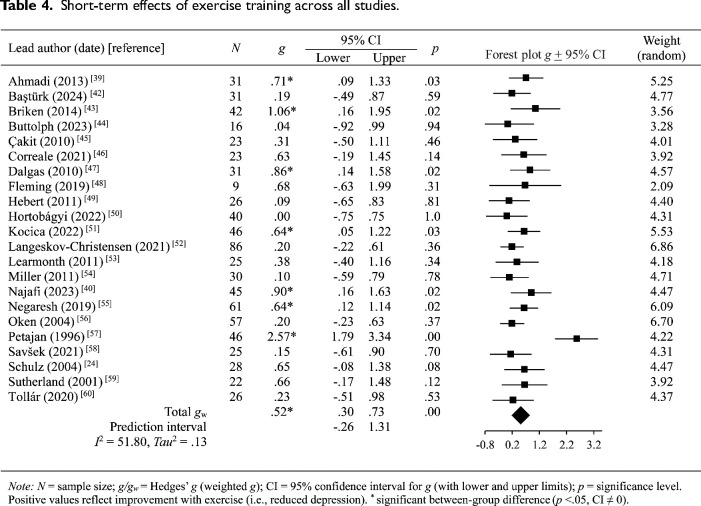
Short-term effects of exercise training across all studies.

**Table 5.** Effect sizes grouped by depression measure and assessment time frame.

Assessment time	Measure	*k*	*g/g_w_*	95% CI		Between-study heterogeneity			
				Lower	Upper	PI	*I^2^*	Tau	Tau^2^
Short-term									
(pre to post)	PROMIS	1	.04	−92	.99				
	HADS-D	5	32	−02	.67	–	0.00	.00	.00
	POMS-D/SF	5	1.08*	16	2.00	−2.31–4.47	86.46	.95	.91
	IDS-R_30_	1	1.06*	16	1.97				
	MDI	2	46	−18	1.10	–	59.59	.36	.13
	BDI/BDI II	10	45*	.24	.67	–	0.00	.00	.00
	CES-D	1	23	–21	68				
	QIDS	1	05	−1.18	1.28				
Total *g_w_*		22	52	.36	73	−26–1.31	51.81	.36	.13
Longer-term									
(post to follow-up)	MDI	2	−68*	−1.05	–31	–	0.00	.00	.00
	HADS	1	−52*	−80	25		0.00	.00	.00
	BDI-II	2	00	−43	42	–	0.00	.00	.00
Total *g_w_*		5	−53*	–80	–24	–	0.00	.00	.00

*k*: number of studies contributing to these data; *g/gw*: Hedges’ *g*/weighted *g*; CI: 95% confidence interval for *g* (with lower and upper limits); PI: prediction interval for pooled *g* (note: PI not calculated when there is a common effect). BDI: Beck Depression Inventory (includes second edition); CES-D: Center for Epidemiological Studies Depression; HADS-D: Hospital Anxiety and Depression Scale – Depression subscale; IDS-R_30_: Self-rated Inventory of Depressive Symptomatology; POMS-D: Profile of Mood States – Depression subscale (includes short form); MDI: major depression inventory; QIDS: Quick Inventory of Depressive Symptomatology.

Positive values reflect improved mood (i.e., reduced depression) with exercise.

* Significant between-group difference (*P *< .01, CI ≠ 0).

Within-study effects varied depending on the depression measure (Table 5). As an example, the same group of participants identified improvements in depression with yoga (Profile of Mood States-Depression subscale *g *= 1.59; CI: .16–3.12^48^) – but no significant pre-post difference with a clinical screening tool (Quick Inventory of Depressive Symptomology *g *= .05; CI: −1.18 to 1.28^48^).

Five studies examined the residual effects of exercise on depression symptoms compared to control groups (see Table 5^47,49,52–54^). The pooled effect was negative and significant: between 4 and 24 weeks, the benefits of exercise had gone and depression scores had worsened.

### Moderating role of exercise parameters

Depression symptoms improved regardless of exercise session frequency (*Q_B_* (1) = 3.49, *P *= 0.06), program duration (*Q_model_* (1) = .46, *P *= 0.49; *R*^2 ^= −.05) or the type of activity performed (*Q*_B_ (2) = 1.25, *P *= 0.53; Table 6). Although baseline depression scores did not significantly predict improvement with exercise (*Q_model_* (1) = 3.36, *P *= 0.06; *R*^2 ^= .25), EDSS scores did: those with minimal or no walking impairment (pre-program) reported greater impacts (*Q*_B_ (1) = 4.97, *P *= 0.03). Individual variation in response was, however, evident. Indeed, the prediction intervals for all subgroup analyses suggested that new trials in this area may even show the opposite (i.e., negative) effect.

**Table 6.** Subgroup analyses.

Variable	*k*	*g_w_*	95% CI	Between-study heterogeneity			
				Prediction interval	*I* ^2^	Tau	Tau^2^
*Session frequency*							
<2 sessions per week	14	33	.15–.50*	–	0.00	.00	.00
>3 sessions per week	7	.81	.24–1.36*	−1.04–2.66	77.84	.66	.43
*Exercise* ^a^							
Aerobic	7	.75	.18–1.31*	−1.15–2.65	81.33	.68	.46
Non-aerobic	5	.53	.14–.91*	–19–1.25	7.44	.12	.01
Mixed	6	.46	.16–.76*	–	0.00	.00	.00
*Disability severity*							
EDSS < 5.0	14	.72	.42–1.01*	−29–1.72	63.18	.43	.18
EDSS > 5.0	4	.17	−20–.55	–	0.00	.00	.00

*k*: number of studies contributing to these data; *g_w_*: weighted Hedges’ *g*; CI: 95% confidence interval for *g* (with lower and upper limits).

* Significant between-group difference (*P* < 0.01, CI ≠ 0).

a Four studies that evaluated aerobic and non-aerobic exercise with the same control group were excluded from this analysis.^39,50,56,60^

## Discussion

The present review consolidated data from 22 randomised controlled trials to highlight the positive and immediate effects of supervised exercise training in managing depression symptoms for adults living in the community with multiple sclerosis. This finding is consistent with previous reviews, which confirm the psychological benefits of regular physical exercise for multiple sclerosis.^21,22^ Individual differences in response to a given exercise program were, however, evident. Moreover, the benefits of exercise for depression were not necessarily maintained post-training. These findings highlight the importance of ongoing treatment and monitoring in multiple sclerosis care.^63^

Importantly, most studies included in this review aligned with evidence-based guidelines for exercise prescription, which highlight the importance of a moderate activity workload (e.g., up to 45 min three times per week), with session duration and volume steadily increasing as physical and mental endurance improve (e.g., Refs. ^12,13,25^). Individually tailored programs according to aerobic fitness level^54^ and rest periods between exercise sets^55^ also helped to maximise treatment effects.

The reliance on a group format for exercise training, and the opportunity for socialisation that this affords, is noteworthy. The rewarding effects of communal exercise in the general population are well-established.^64^ The benefits of small-group work for those with multiple sclerosis do, however, depend on individual characteristics.^65^ For example, there is evidence that males who are diagnosed with multiple sclerosis require more support to engage in physical activity^66^ and experience a higher disability trajectory.^67^ Examination of gendered differences is, however, challenging given the limited sample diversity in multiple sclerosis research in general,^68,69^ including an exclusive focus on women (e.g., Refs. ^39,40,46,48^) and those with mild disability (e.g., Refs. ^40,51,55^). Multi-site RCTs involving different demographic and clinical samples are needed to improve current understanding of treatment response to exercise, including how these interventions can be better tailored to individual need and goals.^70^

Further research is also needed to clarify the effectiveness of non-aerobic training methods on multiple sclerosis–related depression. Whilst Pilates-based core stability training is encouraged^12^ and may appeal to a more sedentary population (i.e., older adults^71^), the evidence-base for multiple sclerosis remains preliminary.^20^ The findings of this review suggest that this is an area that warrants additional consideration. Similarly, the use of combination (aerobic and non-aerobic) techniques requires further evaluation, with growing research indicating that more than one approach is necessary to manage depression in multiple sclerosis.^72^

A further consideration is program adherence. Individual studies reported high rates of withdrawal (up to 40%^42,44,48^) and highlighted issues with transport and accessibility (e.g., Refs. ^42,56^). For this reason, technology-based interventions involving live feedback with a therapist may be able to cater to the changing nature of disease symptoms and impairments over time.^40^ Alternatively, incidental exercise (e.g., outdoor-assisted walking) can be used to introduce physical activity and gradually build fitness, although the evidence base for this type of exercise remains sparse for multiple sclerosis.^73^

Our results additionally highlight the importance of selecting a reliable assessment tool when evaluating a complex construct such as depression. For example, whilst the POMS has been widely used to measure transient mood states, the clinical significance of its subscale scores is not clear – particularly among medical groups.^74^ Although the Beck Depression Inventory and Hospital and Anxiety Depression Scales produced more consistent results in the current review, they may still require altered scoring or interpretation given their item overlap with multiple sclerosis symptoms.^75^

Key limitations in the design of studies included in this review must also be factored. Notably, none specifically targeted patients with a confirmed diagnosis of depression. The estimates provided in this review may therefore not reflect the true effects of exercise. Whilst a subgroup analysis involving those that are depressed is one option, such analyses may be underpowered and produce spurious results in a randomised trial. Future research is therefore warranted to evaluate exercise effects with a focus on depression as a primary outcome. In addition, our operationalisation of exercise intensity was limited to session frequency and duration. Studies did not report or measure other parameters in a consistent way (e.g., target heart rate, activity log, exertion rating). Future research can build on this work by using common vocabulary to describe exercise parameters,^25,70,76,77^ as well as a detailed description of intervention elements, potentially guided by exercise-specific tools such as the TESTEX^30^ and PICOT framework.^76^ Finally, the included studies were characterised by detection and performance bias due to challenges in blinding patients and assessors when evaluating exercise as an active intervention. Future research might consider incorporating some assessment of the person's expectations (e.g., expectations of improvement on well-being) to help verify whether noted gains reflect a positive placebo effect.^78^

Nonetheless, our findings highlight that supervised exercise has a positive impact on depressive symptoms in multiple sclerosis. The next steps are to explore the relative advantages of various intensity levels and delivery methods with a diverse patient sample. Doing so will help us better tailor exercise to meet individual need and, ultimately, help to maintain levels of engagement and maximise treatment effectiveness.

**Clinical messages**

- Supervised exercise can help to alleviate depression symptoms in multiple sclerosis.
- The social aspects of exercising in small groups can improve exercise impact.
- The potential benefits of low-intensity non-aerobic exercise, such as yoga and Pilates, require further evaluation.
- The optimal type, volume and intensity of exercise ultimately depends on individual need, goals, and baseline function.

## Supplemental Material

sj-docx-1-cre-10.1177_02692155241262884 - Supplemental material for Best practice exercise for emerging depression in multiple sclerosis: A systematic review and meta-analysis Supplemental material, sj-docx-1-cre-10.1177_02692155241262884 for Best practice exercise for emerging depression in multiple sclerosis: A systematic review and meta-analysis by Kristiina Ahola, Diana Dorstyn and Nicole Prideaux in Clinical Rehabilitation
