# Supplementary material for: Best practice exercise for emerging depression in multiple sclerosis: A systematic review and meta-analysis
Source: Clin Rehabil. 2024 Jul 25;38(9):1171–87. doi: 10.1177/02692155241262884 (PMC11468659; doi:10.1177/02692155241262884)
Supplement: sj-docx-1-cre-10.1177_02692155241262884 - Supplemental material for Best practice exercise for emerging depression in multiple sclerosis: A systematic review and meta-analysis [file sj-docx-1-cre-10.1177_02692155241262884.docx]

Online Supplementary Material

Table S1. Logic grids for electronic databases (no date or language filters applied)

| Database | Exercise | Multiple Sclerosis |
| --- | --- | --- |
| *Cochrane Library*    [note: the ‘title, abstract, keyword’, ‘in trials’, and ‘search word variations’ options were selected.] | exercise  human physical conditioning  physical activity  endurance training  resistance training  high-intensity interval training  athletic performance  movement therapy  occupational therapy  physical exertion  physiotherapy  physical therapy | multiple sclerosis  demyelinating autoimmune disease  demyelinating disease  demyelinating disorder  disseminated sclerosis |
| *Embase*  [note: ‘include related terms’ was selected.] | exp exercise  (exercise OR exercise training OR fitness training OR fitness workout OR physical exercise OR physical exertion OR physical workout OR aerobic exercise OR anaerobic exercise OR aquatic exercise OR calisthenics OR circuit training OR cross training OR dynamic exercise OR endurance training OR gymnastics OR high intensity exercise OR interval training OR low intensity exercise OR moderate intensity exercise OR pilates OR plyometrics OR power training OR resistance training OR physiotherapy OR physical therapy).ti,ab  physiotherapy.sh | exp multiple sclerosis  (multiple sclerosis OR disseminated sclerosis OR MS OR multiple sclerosis, chronic progressive OR multiple sclerosis, relapsing-remitting OR primary progressive, multiple sclerosis OR relapsing remitting multiple sclerosis OR sclerosis, disseminated OR sclerosis, multiple OR secondary progressive multiple sclerosis OR demyelinating disease OR demyelination).ti,ab |
| *PEDro*  [note: advanced search option used, and “exercise AND multiple sclerosis” inserted into the ‘Abstract and title’ search option. The ‘clinical trial’ option was selected for the ‘method’ category.] | Exercise | multiple sclerosis |

| Database | Exercise | Multiple Sclerosis |
| --- | --- | --- |
| *PsycINFO*  [note: the ‘include related terms’ filter was selected.] | exp exercise  exercis*.mp  movement therap*.mp  movement therapy.sh  physical activit*.mp  physical activity.sh  athletic performance.sh  athletic performance.mp  athletic training.mp  athletic training.sh  exp occupational therapy  occupational therap*.mp  physical therap*.mp  physical therapy.sh  exp physical endurance  physical endurance.mp  physical exertion.mp  exp physical therapy  physical therap*.mp  physiotherap*.mp | exp multiple sclerosis  multiple sclerosis.mp  disseminated sclerosis.mp  demyelinating disorder*.mp  demyelinating disease*.mp  exp demyelination  demyelinating autoimmune disease*.mp |
| *PubMed*  [note: advanced search option used, applying the ‘clinical trials’ filter.] | “exercise”[mh]  exercis*[tiab]  physical conditioning[tiab]  resistance training[tiab]  “athletic performance”[mh]  athletic performance[tiab]  physical endurance[tiab]  physical activit*[tiab]  “physical exertion”[mh]  physical exertion[tiab]  “recreation therapy”[mh]  recreation therap*[tiab]  “physical therapy modalities”[mh:noexp]  “exercise therapy”[mh]  physical therap*[tiab]  endurance training[tiab]  resistance training[tiab] | “multiple sclerosis”[mh]  multiple sclerosis[tiab]  “demyelinating diseases”[mh:noexp]  “demyelinating autoimmune diseases, CNS”[mh]  demyelinat*[tiab]  demyelinating disorder[tiab]  demyelinating disease[tiab] |
